# TBC-2 Is Required for Embryonic Yolk Protein Storage and Larval Survival during L1 Diapause in *Caenorhabditis elegans*

**DOI:** 10.1371/journal.pone.0015662

**Published:** 2010-12-28

**Authors:** Laëtitia Chotard, Olga Skorobogata, Marc-André Sylvain, Sanhita Shrivastava, Christian E. Rocheleau

**Affiliations:** Division of Endocrinology and Metabolism, Department of Medicine, Royal Victoria Hospital, McGill University Health Centre Research Institute, McGill University, Montreal, Quebec, Canada; Brown University, United States of America

## Abstract

*C. elegans* first stage (L1) larvae hatched in the absence of food, arrest development and enter an L1 diapause, whereby they can survive starvation for several weeks. The physiological and metabolic requirements for survival during L1 diapause are poorly understood. However, yolk, a cholesterol binding/transport protein, has been suggested to serve as an energy source. Here, we demonstrate that *C. elegans* TBC-2, a RAB-5 GTPase Activating Protein (GAP) involved in early-to-late endosome transition, is important for yolk protein storage during embryogenesis and for L1 survival during starvation. We found during embryogenesis, that a yolk::green fluorescent protein fusion (YP170::GFP), disappeared much more quickly in *tbc-2* mutant embryos as compared with wild-type control embryos. The premature disappearance of YP170::GFP in *tbc-2* mutants is likely due to premature degradation in the lysosomes as we found that YP170::GFP showed increased colocalization with Lysotracker Red, a marker for acidic compartments. Furthermore, YP170::GFP disappearance in *tbc-2* mutants required RAB-7, a regulator of endosome to lysosome trafficking. Although *tbc-2* is not essential in fed animals, we discovered that *tbc-2* mutant L1 larvae have strongly reduced survival when hatched in the absence of food. We show that *tbc-2* mutant larvae are not defective in maintaining L1 diapause and that mutants defective in yolk uptake, *rme-1* and *rme-6*, also had strongly reduced L1 survival when hatched in the absence of food. Our findings demonstrate that TBC-2 is required for yolk protein storage during embryonic development and provide strong correlative data indicating that yolk constitutes an important energy source for larval survival during L1 diapause.

## Introduction

In many oviparous species, yolk proteins or vitellogenins, and their associated lipids, are an important food source for developing embryos [1]. *Caenorhabditis elegans* vitellogenins have homology with human ApoB-100, the primary component of low-density lipoprotein (LDL) [2]; [3], and like ApoB-100, they mediate cholesterol transport [4]. *C. elegans* YP170, YP115 and YP88 yolk proteins are synthesized in hermaphrodite intestine and secreted into the body cavity [5]. Yolk protein is then internalized into maturing oocytes via receptor-mediated endocytosis of the RME-2/LDL receptor where it resides in puncta or vesicles [6]. It has been shown in other organisms that internalized yolk is stored in yolk granules or yolk platelets, lysosomal-like compartments with a neutral or mildly acidic pH, and that regulated acidification can activate latent proteases such as cathepsin L to control yolk degradation [1]. During *C. elegans* embryogenesis, yolk granules are present in the blastomeres of the dividing embryo, and during morphogenesis yolk is transferred into the intestinal cells [7].

Yolk protein trafficking can be followed using a yolk protein::green fluorescent protein fusion (YP170::GFP) which has been used to identify genes required for yolk uptake and trafficking such as *rme-2* [6]. Mutations in *rme-2*, the yolk receptor/LDLR homolog, block YP170::GFP internalization, display slow growth and partial embryonic lethal phenotypes. Mutations in other *rme* genes identify endocytic regulators such as *rme-1*, a conserved EH-domain protein required for endosome recycling, and *rme-6*, a RAB-5 Guanine nucleotide Exchange Factor (GEF) [8]; [9]. Despite having strongly reduced YP170::GFP uptake, *rme-1(b1045)* and *rme-6(b1014)* embryos are viable. The absolute requirements for yolk proteins during *C. elegans* development remains unknown, due in part to the fact that there are six genes coding for vitellogenins.

When L1 larvae hatch in absence of food, they enter an “L1 diapause” or developmental arrest [10]. These arrested animals can survive for a few weeks under starvation conditions without changing morphology and can resume larval development when reintroduced to food without affecting lifespan. Since yolk has been detected in the intestine of hatching larvae, it has been suggested that yolk, in addition to serving as an energy source during embryogenesis, could serve as an energy source for larvae that hatch in the absence of food [7].

TBC-2 is a RAB-5 GAP that regulates endosome and phagosome maturation [11]; [12]. In *tbc-2* mutant oocytes, YP170::GFP accumulates in enlarged granules/vesicles [11]. Here, we follow the fate of YP170::GFP during embryonic development in *tbc-2(tm2241)* mutants. We found that YP170::GFP colocalized with Lysotracker Red, a marker for lysosomes, and was rapidly degraded. The localization and degradation of YP170::GFP in *tbc-2(tm2241)* embryos was RAB-7 dependent, consistent with TBC-2 antagonizing Rab GTPase activity. Despite the rapid depletion of yolk protein, *tbc-2(tm2241)* embryos are viable. However, we found that *tbc-2(tm2241)* L1 larvae had reduced survival under starvation conditions. This reduced survival is not due to a defect in entering or maintaining L1 diapause, but may be due to reduced yolk stores, as other mutants with reduced YP170::GFP uptake, *rme-1* and *rme-6*, were similarly compromised for L1 survival during starvation conditions.

## Results

### Yolk protein YP170 is prematurely lost in *tbc-2(tm2241)* embryos

**Figure 1:**
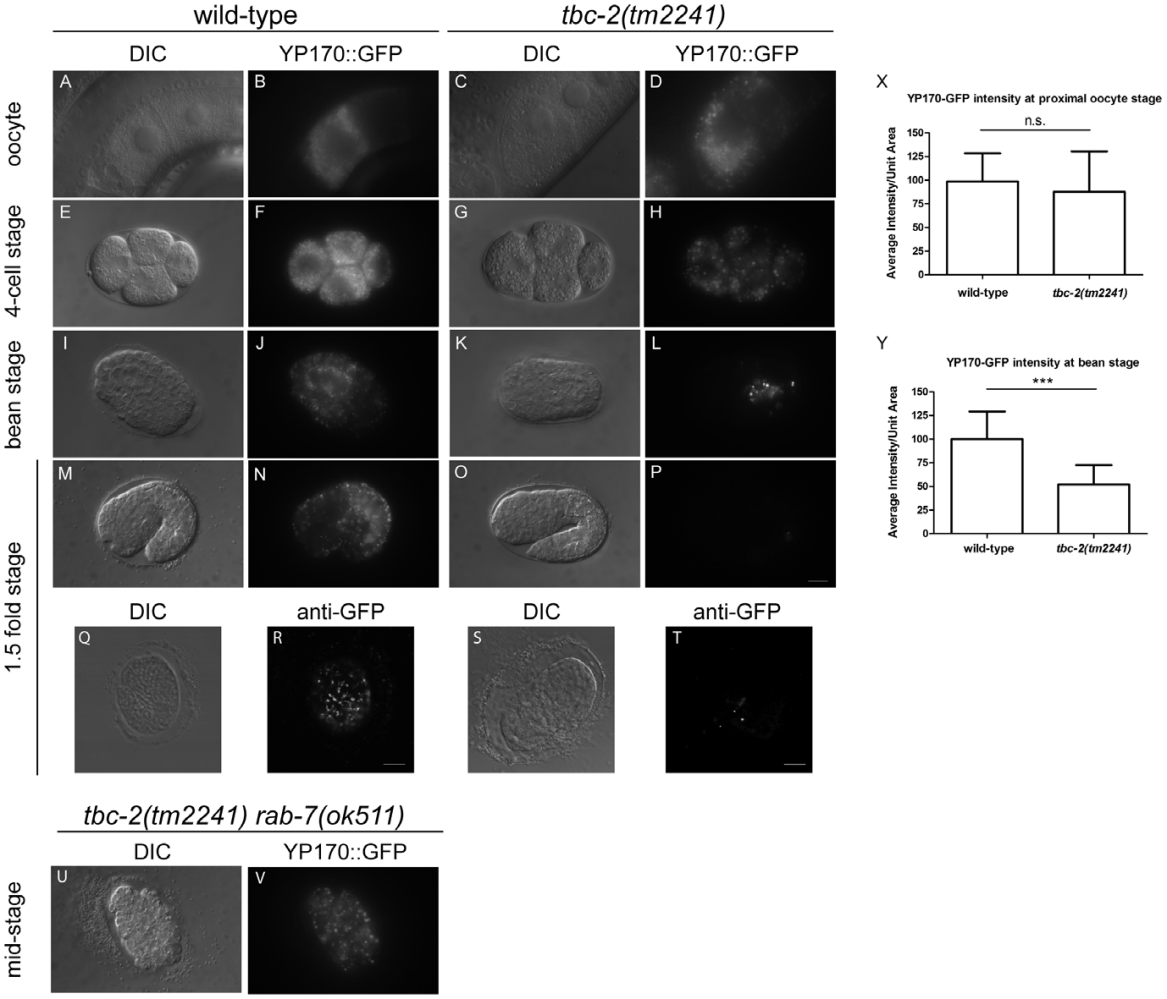
Premature depletion of YP170::GFP in *tbc-2(tm2241)* embryos. Differential Interference Contrast (DIC) (A, C, E, G, I, K, M, O, Q, S and U) and epifluorescence (B, D, F, H, J, L, N, P, R, T and V) images of wild-type (A, B, E, F, I, J, M, N, Q and R), *tbc-2(tm2241)* (C, D, G, H, K, L, O, P, S and T), and *tbc-2(tm2241) rab-7(ok511)* (U and V) oocytes (A–D) and different stage embryos (4-cell, E-H; bean stage at the beginning of morphogenesis, I-L; 1.5 fold stage of elongation, M-T; mid-stage of nonviable *tbc-2(tm2241) rab-7(ok511)* is late proliferative/early morphogenesis stage, U and V) carrying maternally deposited YP170::GFP from the integrated transgene *bIs1*. All images are of live animals except Q-T, which are fixed and immunostained with an anti-GFP antibody. Quantification of YP170::GFP fluorescence average intensity per unit area in wild-type and *tbc-2(tm2241)* proximal oocytes and bean stage embryos (*n* = 23 for both strains) (X and Y). A two-tailed unpaired Student t-test was used to determine statistical significance. n.s., not significant. ***, p<0.0001. Error bars represent standard deviations. Bars, 10 µm.

YP170::GFP is internalized into maturing oocytes in both wild-type and *tbc-2(-)* animals, however YP170::GFP granules are larger in *tbc-2(-)* oocytes and early embryos (Fig. 1A-1D; [11]). We quantified the levels of YP170::GFP fluorescence in oocytes of wild-type and *tbc-2(tm2241)* mutants and found no significant difference (Fig. 1X), consistent with *tbc-2(tm2241)* animals not having significant defects in yolk synthesis, secretion, or uptake. To determine if *tbc-2(-)* embryos are defective in the trafficking or storage of yolk, we followed the localization of YP170::GFP during later stages of embryonic development and found that the YP170::GFP fluorescence was strongly reduced in *tbc-2(tm2241)* embryos as compared to similarly staged wild-type embryos (Fig. 1E-1P). We start to see a decrease in YP170::GFP fluorescence as early as the 4-cell stage of embryogenesis (Fig. 1E-1H), and by the bean stage, prior to elongation, YP170::GFP fluorescence is reduced by 50% in *tbc-2(tm2241)* embryos as compared to wild-type (Fig. 1I-1L, 1Y). By the 1.5 fold stage, very little fluorescence is evident in *tbc-2(tm2241)* embryos (Fig. 1M-1P). We see the same loss of YP170::GFP by immunostaining with an anti-GFP antibody (Fig. 1Q-1T), suggesting that YP170::GFP is prematurely degraded in *tbc-2(tm2241)* embryos.

### YP170 is targeted to lysosomes in a RAB-7-dependent manner in *tbc-2(-)* embryos

**Figure 2:**
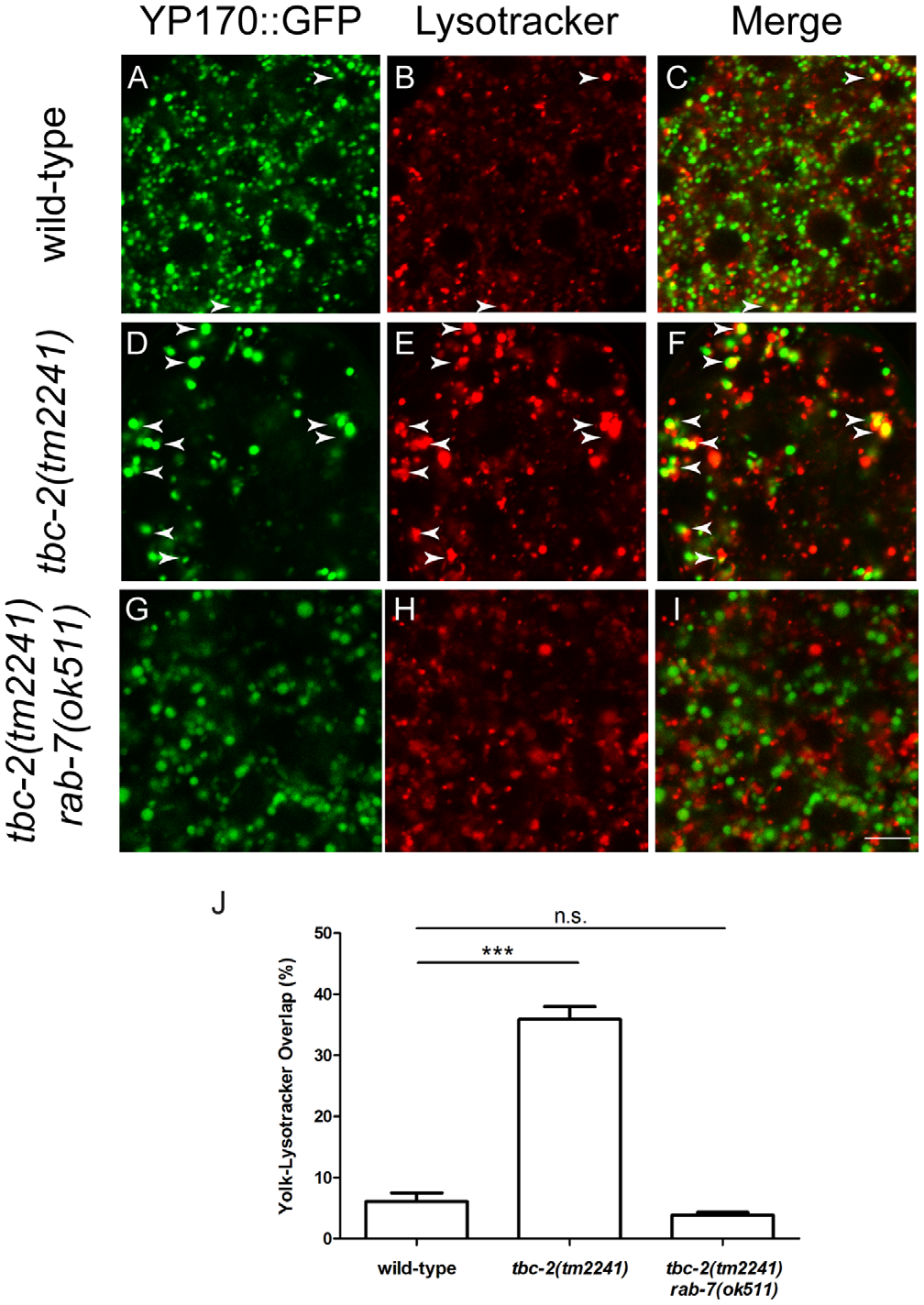
YP170::GFP localizes to lysosomes in *tbc-2(tm2241)* embryos. Confocal images of wild-type (A–C), *tbc-2(tm2241)* (D–F), and *tbc-2(tm2241) rab-7(ok511)* (G–I) embryos carrying YP170::GFP (green; A, D, and G) and stained with Lysotracker Red (B, E, and H) with the colocalization shown in the Merge images (C, F, and I). Arrows mark colocalization between YP170::GFP vesicles and Lysotracker Red. Quantification of the percentage of YP170::GFP fluorescence overlapping with Lysotracker Red compared to the total YP170::GFP fluorescence (J). *n = *25, wild-type; 17, *tbc-2(tm2241);* 10, *tbc-2(tm2241) rab-7(ok511)*. Statistical significance was determined using a two-tailed unpaired Student t-test. n.s., not significant. ***, p<0.0001. Error bars represent standard deviations. Bar, 5 µm.

Consistent with yolk being stored in distinct lysosomal-related compartments, YP170::GFP does not significantly colocalize with Lysotracker Red, a marker for acidic lysosomes in wild-type embryos (Fig. 2A-2C). TBC-2 regulates endosomal trafficking, therefore we hypothesized that yolk granules are prematurely acidified or yolk may be mistargeted to lysosomes in *tbc-2(-)* embryos. Consistent with this hypothesis, there was a higher incidence of colocalization between YP170::GFP and Lysotracker Red in *tbc-2(tm2241)* embryos (Fig. 2D-2F, 2J), suggesting that YP170::GFP is being prematurely degraded in the lysosomes of *tbc-2(tm2241)* embryos.

Rab7 mediates endosome to lysosome trafficking [13]-[16], therefore, we tested whether RAB-7 was required for YP170::GFP degradation in *tbc-2(-)* embryos. We found that YP170::GFP persisted in embryos of *tbc-2(tm2241) rab-7(ok511)* double mutants (Fig. 1U and 1V), and almost no colocalization of YP170::GFP with Lysotracker Red was seen in *tbc-2(tm2241) rab-7(ok511)* double mutants (Fig. 2G-2J), indicating that RAB-7 is required for the lysosomal trafficking and degradation of YP170::GFP in *tbc-2(tm2241)* embryos.

### *tbc-2(tm2241)* animals display reduced survival during starvation-induced L1 diapause

**Figure 3:**
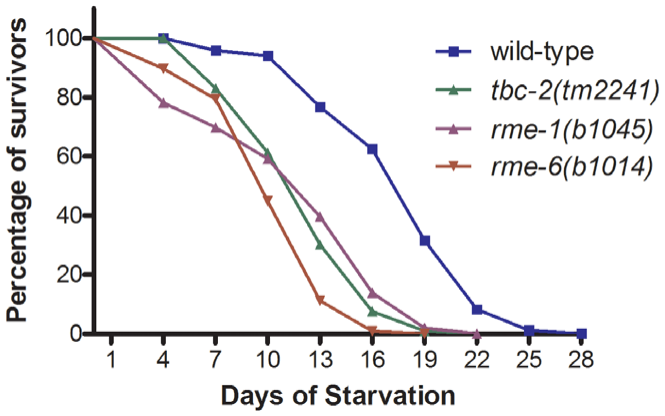
*tbc-2(tm2241), rme-1(b1045),* and *rme-6(b1014)* L1 larvae have reduced survival during starvation. Survival curve of wild-type (blue), *tbc-2(tm2241)* (green), *rme-1(b1045)* (purple), and *rme-6(b1014)* (red) L1 larvae hatched in the absence of food. Graph represents the average of three independent experiments.

Under normal laboratory culture conditions, *tbc-2(-)* animals do not show overt defects in development or fertility, suggesting that the reduced yolk protein in *tbc-2(-)* animals does not have any deleterious effects when there is an abundance of food. Animals that hatch in the absence of food enter into an L1 diapause or developmental arrest and can survive for several weeks. Yolk stored in the gut of hatching L1s has been proposed to serve as an energy source during L1 diapause as it has been observed that yolk disappears in L1 larvae after starvation [7]. Therefore we tested whether *tbc-2(tm2241)* larvae display reduced survival when hatched in the absence of food. We found that *tbc-2(tm2241)* larvae hatched in the absence of food have a significantly reduced survival with a mean survival of 13 days, as compared to wild-type controls with mean survival of 19 days (*P*<0.0001) (Fig. 3). Thus, the survival of *tbc-2(tm2241)* larvae hatched in the absence of food is compromised.

### Starved *tbc-2(tm2241)* larvae are not defective in maintaining L1 diapause

**Figure 4:**
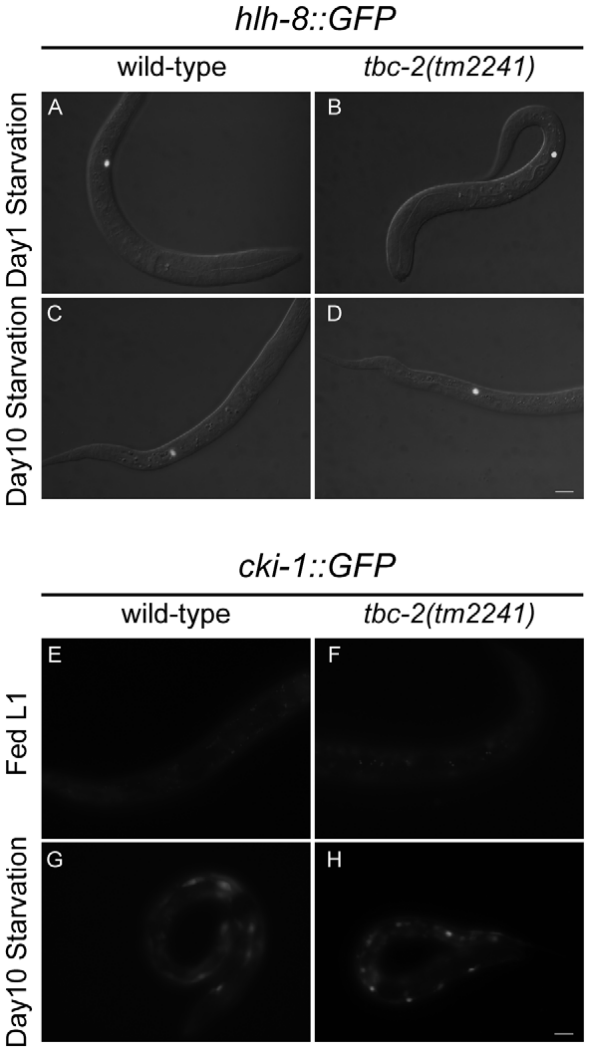
*tbc-2* is not required for maintaining L1 starvation-induced diapause. (A–D) Merged DIC and epifluorescence images of *hlh-8::GFP* expression in the M cell of wild-type (A and C) and *tbc-2(tm2241)* (B and D) L1 larvae at day 1 (A and B) and day 10 (C and D) of starvation. (E–H) Epifluorescence images of *cki-1::GFP* expression in wild-type (E and G) and *tbc-2(tm2241)* (F and H) in fed L1 larvae (E and F) and L1 larvae at day 10 of starvation (G and H). Bar, 10 µm.

A possible explanation for the reduced survival of *tbc-2(tm2241)* animals during starvation could be a failure to initiate or maintain L1 diapause in the absence of food. To assess whether *tbc-2(tm2241)* larvae were defective in developmental arrest, we followed the lineage of the M blast cell in day 1 and day 10 starved L1 larvae. The M blast cell gives rise to mesodermal tissues and normally divides several hours into the L1 stage in fed larvae [17] and can be easily followed with an *hlh-8::GFP* marker [18]; [19]. We found that the M blast cell never divided in starved wild-type (*n = *115 day 1, *n = *173 day 10) or *tbc-2(tm2241)* (*n* = 116 day 1, *n* = 172 day 10) larvae at either time point (Fig. 4A-4D).

The expression of the cell cycle inhibitor, *cki-1*, is upregulated in the seam cells of starved L1 larvae and low in fed larvae as assessed with the transcriptional reporter, *cki-1::GFP* [20]; [21]. Similarly, we found that *cki-1::GFP* expression was low in fed wild-type and *tbc-2(tm2241)* larvae and upregulated in the seam cells of day 10 starved wild-type and *tbc-2(tm2241)* larvae. (Fig. 4E-4H). Together, these data indicated that *tbc-2(tm2241)* larvae arrest development and maintain L1 diapause during starvation.

### *rme-1* and *rme-6* mutants have reduced survival during starvation-induced L1 diapause

If the reduced L1 survival of starved *tbc-2(tm2241)* larvae is due to having reduced levels of yolk, then we would predict that other viable mutants with reduced yolk should also have reduced L1 survival during starvation. Therefore we measured the survival of *rme-1(b1045)* and *rme-6(b1014)* mutants, which like *tbc-2(tm2241)* are viable and have strongly reduced embryonic YP170::GFP expression. We found that *rme-1(b1045)* and *rme-6(b1014)* L1 larvae hatched in the absence of food also have significantly reduced mean survival of 13 days (*P*<0.0001) and 10 days (*P*<0.0001) respectively, as compared to wild-type (19 days), and similar to that of *tbc-2(tm2241)* (13 days). These findings are consistent with yolk having an important role in L1 survival during starvation conditions.

## Discussion

*C. elegans* yolk protein trafficking in oocytes requires the activities of RAB-5 and RME-6 (the RAB-5 GEF, an activator of RAB-5) for endocytosis/internalization and RAB-7 for movement away from the cell periphery [6]; [9]. We have recently demonstrated a role for TBC-2 as a RAB-5 GAP and a negative regulator of Rab GTPase-mediate endosome trafficking [11]. In addition to having enlarged yolk vesicles in oocytes, *tbc-2(-)* animals have enlarged endosomes in coelomocytes and intestinal cells. The large late endosome phenotype in the intestine requires the activities of both RAB-5 and RAB-7, consistent with the phenotypes being due to increased Rab GTPase activity. As with the intestinal phenotype, the premature disappearance of yolk protein in *tbc-2(tm2241)* embryos is RAB-7-dependent. While it is not known how internalized yolk comes to be in specialized yolk granules/platelets, our data indicate that TBC-2 is required either for the sorting of yolk into yolk granules or for the stability/maintenance of yolk granules. In the later case, TBC-2 could be required for the sorting of a critical component of yolk granules, preventing premature acidification or fusion with lysosomes. Since Rab7 mediates fusion of late endosomes with lysosomes in mammalian cells [22]; [16] and the yeast Rab7, Ypt7p mediates homotypic fusion of vacuoles [23], we would predict that too much RAB-7 activity drives fusion between yolk granules and lysosomes in *tbc-2(tm2241)* mutants, but further analysis will be required to determine the molecular mechanisms involved.

While yolk serves as a food source for the embryo in egg laying species, the full requirements for yolk in *C. elegans* is not known. Since mutations in the yolk receptor, *rme-2*, are partially lethal, it would suggest that yolk is important for embryonic development, but it is also possible that RME-2 has additional requirements during embryogenesis [6]. Other genes that regulate yolk trafficking, such as *rab-5, rab-7,* and *cup-5* (a late endocytic regulator), are embryonic lethal, however this lethality is probably not specific to defects in yolk trafficking [24]; [6]. *cpl-1* (a cathepsin L cysteine protease) mutants are embryonic lethal and defective in yolk processing, but lethality may be due to yolk platelet aggregation in the cytoplasm rather than an energetic requirement for yolk [25]. Meanwhile, mutations in *rme-1* and *rme-6* have strongly reduced yolk uptake, yet are viable [8]; [9]. Bossinger and Schierenberg (1996) hypothesize that yolk present in the intestine of hatching larvae might be important for animal survival. Our data showing that *tbc-2(-), rme-1(-)* and *rme-6(-)* larvae have reduced survival during starvation-induced L1 diapause support this hypothesis, but do not preclude a role for yolk during embryogenesis or that these genes could regulate survival by other means. Assuming that the reduced survival is due to depletion of energetic resources, these endocytic regulators could affect other processes required for survival such as autophagy or insulin/IGF signaling. Our results show that *tbc-2*, *rme-1*, and *rme-6*, while not essential under standard laboratory conditions, are likely important for survival in the wild.

## Materials and Methods

### *C. elegans* alleles and general methods

General methods for the handling and culturing *C. elegans* were as previously described [26]. *C. elegans var* Bristol strain N2 is the wild-type parent for all strains used in this work. *E. coli* strain HB101 was used as a food source. Specific genes and alleles are described on Wormbase (www.wormbase.org) and are available from the Caenorhabditis Genetics Center. LGII: *tbc-2(tm2241), rab-7(ok511)*; LGV: *rme-1(b1045)*; LGX: *rme-6(1014), bIs1[vit-2::GFP + rol-6(su1006)];* Linkage unknown: *maIs113[cki-1::GFP + dpy-20(+)]; ayIs7 [hlh-8::GFP + dpy-20(+)]*.

### Microscopy and Phenotype Analysis

General methods for Nomarski differential interference contrast (DIC) microscopy of live animals were as previously described [17]. Animals were analyzed on an Axio Zeiss A1 Imager compound microscope (Zeiss, Oberkochen, Germany) and images were captured using an Axio Cam MRm camera and AxioVision software (Zeiss, Oberkochen, Germany). Comparison of YP170::GFP expression in wild-type and *tbc-2(tm2241)* embryos was performed using identical exposure times and image modifications for each embryonic stage. Oocyte images were taken with reduced exposure.

Confocal analysis was performed using a Zeiss LSM-510 Meta laser scanning microscope with 63X oil immersion lens in a multi-track mode using a single or dual excitation (488 nm for GFP and/or 543 nm for mCherry). Images were captured using LSM Image software (Zeiss, Oberkochen, Germany). Immunostaining of YP170::GFP in *C. elegans* embryos was carried out using the freeze-crack method and methanol/acetone fixation [27]. A Goat anti-GFP antibody (Rockland Inc., Gilbertsville, PA) was used at a 1:100 dilution and a secondary rabbit anti-goat antibody conjugated to AlexaFluor 488 (Invitrogen, Carlsbad, CA) was used at a 1∶200 dilution. Lysotracker Red staining was performed as previously described [24]. NGM plates were complemented with Lysotracker Red (Invitrogen, Carlsbad, CA) at a concentration of 50 nM, and then plates were seeded with HB101 bacteria containing 2 µM Lysotracker Red. Quantification of fluorescence and colocalization images was performed using Metamorph (Universal Imaging Corp.). Statistical analysis and graphing was done using Prism 5 (GraphPad Software, Inc., La Jolla, CA).

### L1 Survival Assays

Starvation survival assays were performed as previously described [28]. Synchronized L1 larvae were incubated in 3 mL of sterilized M9 buffer and animals were kept at 20°C. In triplicate, 20 µL aliquots from each sample were placed on individual seeded plates every 3 days. The number of survivors was determined after 3 days at 20°C. Day 1 is considered to be the first day of starvation and was used as the 100% survival point to calculate the percentage of survivors for the proceeding time points. Results are derived from at least three independent experiments. The average percent survival for each time point of the three independent experiments was determined to derive the survival curve in Fig. 3. Statistical analysis and graphing was done using Prism 5 (GraphPad Software, Inc., La Jolla, CA). Log-rank (Mantel-Cox) test was used to compare the percentage of survivors and create survival curves for each strain.
